# Biochemical, Clinical, and Functional Characterization of a Rare c.‐106C>A Promoter Region Variant in Late‐Onset Ornithine Transcarbamylase Deficiency: A Multifamily Case Series

**DOI:** 10.1002/jmd2.70064

**Published:** 2026-01-15

**Authors:** Samuel Quinn Tholl, Wendy McCaul, Anthony Rupar, Melanie P. Napier, Natalya Karp, Andrea C. Yu, Suzanne Ratko, Aneal Khan, Michael Geraghty, Chitra Prasad

**Affiliations:** ^1^ Western University London Ontario Canada; ^2^ Department of Pathology and Biochemistry London Health Sciences Centre London Ontario Canada; ^3^ Department of Paediatrics London Health Sciences Centre London Ontario Canada; ^4^ GeneDx Gaithersburg Maryland USA; ^5^ Children's Hospital of Eastern Ontario Ottawa Ontario Canada; ^6^ Cumming School of Medicine University of Calgary Calgary Alberta Canada; ^7^ M.A.G.I.C. (Metabolics and Genetics in Canada) Clinic Calgary Alberta Canada

**Keywords:** hyperammonemia, late‐onset OTC deficiency, *OTC* gene promoter region, urea cycle disorder, X‐linked inheritance

## Abstract

Ornithine transcarbamylase (OTC) deficiency can present during the neonatal period, infancy, or adulthood. Late‐onset phenotypes are influenced by residual enzyme activity and *OTC* gene expression. The objective of this study was to assess the pathogenicity of a rare promoter region variant, c.‐106C>A. We reviewed three independent pedigrees harboring the c.‐106C>A variant. Retrospective chart reviews were conducted for 16 affected males to evaluate biochemical and clinical features. Functional OTC enzyme data were obtained from the liver biopsy of three affected males. The median age of diagnosis for symptomatic males was 15 years (range: 0.08–55). All 21 heterozygous females have been asymptomatic to date. Of the 16 hemizygous males, seven have experienced at least one episode of hyperammonemia, and eight have remained asymptomatic. The proband from Family A presented at age 55, with a peak ammonia level of 694 μmol/L. His OTC activity was 8.2 μmol/g liver/min (control: 94.9 μmol/g liver/min). The proband from Family B presented at age 15 with an ammonia level of 1568 μmol/L and later died from cerebral edema. His OTC activity was 2.2 μmol/g liver/min (control: 73.2 μmol/g liver/min). The proband from Family C presented at age 24 with hyperammonemia and later died following withdrawal of care. His liver punch biopsy OTC enzyme activity was measured to be 0 μmol/g liver/min (control range: 25–31.7 μmol/g liver/min). For most affected males, ammonia scavenger therapy has successfully prevented recurrent decompensation. The combined biochemical, clinical, and enzymatic data strongly support the pathogenicity of the c.‐106C>A *OTC* promoter region variant in late‐onset OTC deficiency.

## Key Points

- This study highlights a rare promoter region variant in the *OTC* gene (c.‐106C>A) that is associated with late‐onset ornithine transcarbamylase deficiency across three independent pedigrees.
- Affected hemizygous males demonstrated marked clinical and biochemical heterogeneity, ranging from asymptomatic status to fatal hyperammonemic crises.
- Liver biopsy data showed severely reduced or absent OTC enzyme activity, supporting a functional impact of the c.‐106C>A promoter variant on gene expression.
- Our findings emphasize the importance of sequencing regulatory regions of the *OTC* gene in suspected cases when coding‐region analysis is initially unrevealing.

## 1 Introduction

Ornithine transcarbamylase (OTC) deficiency (OMIM #311250) is the most common urea cycle disorder (UCD), affecting approximately 1 in 56 000 to 1 in 113 000 individuals [1]. It results from pathogenic variants in the *OTC* gene that impair urea cycle function, leading to the accumulation of ammonia in the body [2]. Without prompt treatment, hyperammonemic episodes can cause cerebral edema, encephalopathy, coma, and ultimately death [3, 4].

OTC deficiency can occur in two main forms [2]. The classical form typically presents in the neonatal period and is caused by a complete loss of OTC function [5]. In contrast, late‐onset OTC deficiency can manifest anytime from infancy to adulthood and is usually triggered by a catabolic stressor, such as increased protein intake, recurrent infections, or metabolic demand [3, 6, 7, 8, 9]. Due to the X‐linked inheritance pattern of OTC deficiency, hemizygous males are disproportionately affected and often present in the neonatal period [2]. However, males with hypomorphic variants may retain partial enzyme activity and develop late‐onset disease [10]. The disease severity for heterozygous females can be influenced by the pattern of lyonization in hepatocytes [2, 11]. Recent data demonstrates that heterozygous carriers may exhibit varied late‐onset manifestations thought to be due to their OTC carrier status, emphasizing the importance of recognizing potential clinical variability in this population [12].

In this paper, we present biochemical, clinical, and enzymatic data from affected males in three independent pedigrees carrying a rare *OTC* gene promoter region variant, c.‐106C>A (NM_000531.5:c.‐106C>A), associated with late‐onset OTC deficiency. We highlight the variable clinical presentations among affected males and integrate our findings with those of Jang et al. and Han et al. to further support the pathogenicity of this variant [10, 13].

## 2 Patients and Methods

Inclusion criteria required individuals to carry the c.‐106C>A variant, have a suspected or confirmed diagnosis of OTC deficiency based on laboratory findings and/or clinical symptoms, or relation to an affected family member. Diagnosis of OTC deficiency may have been confirmed due to OTC activity levels or sequencing of the promoter region of the *OTC* gene. All clinical and biochemical information presented was retrospectively collected and analyzed from patient charts. Based on these criteria, 36 patients overall (21 females, 16 males) from three independent pedigrees containing the c.‐106C>A variant were eligible; however, only the 16 males were included in the study. None of the 21 confirmed heterozygous females experienced any measurable metabolic decompensations, and thus no formal data were collected from them. OTC enzyme activity was also obtained from liver biopsies of three males, including one unlisted member of Family A. Families A and B were seen at a hospital in London, Ontario, and Family C was seen at a hospital in Ottawa, Ontario.

The study protocol was approved by the Research Ethics Board of the University of Western Ontario (UWO) and the Research Ethics Board of Children's Hospital of Eastern Ontario (CHEO).

## 3 Results

### 3.1 Biochemical Characteristics and Clinical Presentations

**FIGURE 1:**
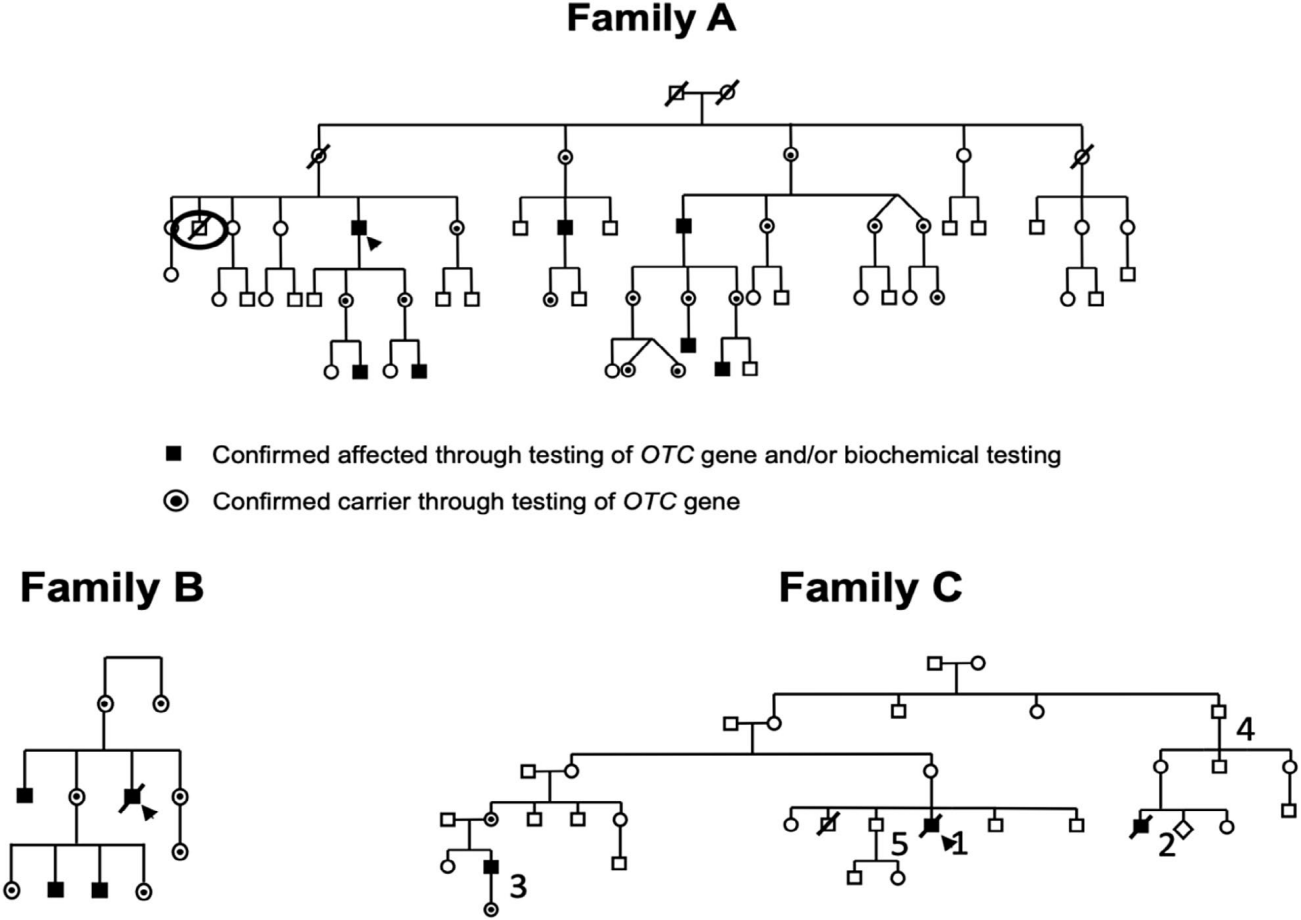
Pedigrees of Family A, Family B, and Family C. Confirmed heterozygous females are marked with a black dot, while hemizygous males are indicated by a solid black square.

The c.‐106C>A variant was identified in three unrelated families. The X‐linked inheritance pattern is evident in the pedigrees of Families A–C (Figure 1). Probands were diagnosed via genetic or OTC enzymatic testing, prompting cascade testing in at‐risk family members. In Families A and C, not all individuals underwent cascade testing. One brother of the Family A proband died decades ago of suspected, though unconfirmed, OTC deficiency (black circle in Figure 1).

Biochemical and clinical data for all affected males are summarized in Table 1. None of the 21 confirmed heterozygous females experienced any measurable metabolic decompensations, though some reported psychiatric issues, recurrent headaches, and migraines, of which we are unsure of the relation to their heterozygosity. The median age of diagnosis was 15 years (IQR: 26.25, range: 0.08–55 years). Nine of the 16 affected males required chronic pharmacological management, and 11 experienced one episode of hyperammonemia, which we defined as an ammonia level > 100 μmol/L [2].

**TABLE 1:** Biochemical, clinical, and familial data for affected males with the c.‐106C>A *OTC* variant from Families A–C.

Indivi‐dual	Relationship to proband	Age/age at diagnosis	Clinical presentation	# Of HAEs	Maximum [ammonia] (15–55 μmol/L)	Maximum [glutamine] (332–754 μmol/L)	[Citrulline] (13.7–63.2 μmol/L)	Maximum [urine orotic acid] (0–7.7 μg/mg creatinine)	Acute treatment	Chronic management	Outcome
*Family A*											
1	Proband for Family 1	71/55	ALC, nausea, vomiting, lethargy, encephalopathy	2	154 694	N/A 1059	N/A 13	N/A N/A	Hemodialysis, sodium phenylacetate and benzoate (ammonul)	Glycerol phenylbutyrate (ravicti), l‐citrulline, protein restrictive diet	Alive
2	Grandson	27/12.5	Asymptomatic	0	48	843	N/A	63	N/A	PRD	Alive
3	Grandson	16/1 month	Asymptomatic	0	71	964	49	10.8	N/A	Glycerol phenylbutyrate (ravicti), l‐citrulline, PRD	Alive
4	Cousin	49/34	Asymptomatic	1	104	799	44	8.4	N/A	Citrulline, sodium phenylbutyrate (pheburane)	Alive
5	Cousin	65/50	Asymptomatic	0	34	592	N/A	7.9	N/A	No treatment	Alive
6	Grand nephew	18/4	Nausea, vomiting	2	379 103	760 1175	17 57	9.3 3.3	Sodium phenylacetate and benzoate	Glycerol phenylbutyrate (ravicti), l‐citrulline, PRD	Alive
7	Grand nephew	18/4.5	Mental health issues	0	82	871	99	30	N/A	Glycerol phenylbutyrate (ravicti), l‐citrulline	Alive
*Family B*											
1	Proband for Family 2	15/15	ALC, lethargy, slurred speech, ataxic gate, anorexia	1	1529	1121	20	891	Hemodialysis, sodium phenylacetate, and benzoate (ammonul), phenytoin, phenobarbiital, hydralazine	Valproate, dexedrine, risperdal, cyproheptadine	Deceased
2	Brother	34/18	Learning disability, mental health issues	0	63	856	59	24.1	N/A	No treatment	Alive
3	Nephew	11/9 months	Learning disability	1	102	971	27	52.4	N/A	Glycerol phenylbutyrate (ravicti), l‐citrulline, PRD	Alive
4	Nephew	9/1 month	Asymptomatic	1	121	881	17	N/A	N/A	Sodium phenylbutyrate (pheburane), citrulline	Alive
*Family C*											
1	Proband for Family 3	14	Fatigue, lethargy, dizziness, confusion, ataxia	1	> 1000	1006	N/A	N/A	No treatment	Self‐restricted vegetarian diet	Deceased
2	Maternal second cousin	24	Encephalopathy	1	1400	N/A	N/A	N/A	No treatment	Self‐restricted vegetarian diet	Deceased
3	Maternal second cousin once removed	30	Asymptomatic	0	39	861	25	19.7	Continue to monitor	Continue to monitor	Alive
4	Maternal great‐uncle	80's	Asymptomatic	0	N/A	N/A	N/A	N/A	N/A	N/A	Unknown
5	Brother	31	Asymptomatic	0	N/A	N/A	N/A	N/A	Continue to monitor	Continue to monitor	Alive

Abbreviations: ALC = altered level of consciousness, HAE = hyperammonemic episodes, PRD = protein restrictive diet.

In Family A, three out of the seven affected males experienced hyperammonemia. Six out of seven are managed with ammonia scavengers, a protein restrictive diet, or both. Intra‐family biochemical and clinical variability was evident: Individual 1 presented with hyperammonemia at age 55, while Individual 7 presented at age 4. During individual 1's first hyperammonemia episode, he exhibited behavioral abnormalities and encephalopathy. Serum ammonia testing was subsequently prompted by his nephew's prior OTC diagnosis, revealing a value of 154 μmol/L. Sequencing of the *OTC* gene, including exons, splice sites, and promoter region, confirmed the presence of the c.‐106C>A variant. During his second hyperammonemia episode at age 70, he displayed altered levels of consciousness, nausea, vomiting, lethargy, and encephalopathy. Preceding gastroenteritis and poor medication adherence due to emesis may have contributed. At the time, ammonia and glutamine levels were 694 and 1059 μmol/L, respectively. Hemodialysis was initiated, and a cocktail of ammonia scavengers was administered. Despite being in a coma for a few days, the proband recovered and is currently managed with glycerol phenylbutyrate, l‐citrulline, and a protein‐reduced diet.

In Family B, all females were identified as carriers, and three out of the four affected males experienced at least one hyperammonemic episode. Two also reported learning disabilities, mental health concerns, or both, which may be partially attributable to OTC deficiency. Similar to Family A, clinical outcomes varied widely, from asymptomatic to fatal. The proband's hyperammonemic crisis was previously documented in a 2006 case report [3]. At age 15, he presented with a 6‐day history of lethargy, anorexia, slurred speech, confusion, and an abnormal gait. At the time, he was prescribed valproate for his behavioral problems. Ammonia and glutamine were measured to be 1529 and 1121 μmol/L, respectively, and urine orotic acid was 891 μg/mg creatinine. Despite intense ammonia lowering methods via hemodialysis and ammonia scavenger administration, autonomic instability ensued due to encephalopathy, and imaging showed absent perfusion to the cortex and brainstem. Treatment was subsequently withdrawn as per the family's decision. At the time the c.‐106C>A variant was not detected by sanger sequencing as only exons and intron splice site sequences were analyzed, however an OTC deficiency diagnosis was later confirmed postmortem from a liver biopsy taken before care was withdrawn.

In Family C, Individual 1 was the first to present with hyperammonemia, though genetic testing was unavailable at the time. Years later, Individual 2 was diagnosed with OTC deficiency via liver biopsy, and linkage analysis subsequently confirmed Individuals 2, 4, and 5 shared the same X‐chromosome. The c.‐106C>A variant was later identified as familial, and individual 1 was retrospectively presumed affected based on his medical records in light of the new family history. Two of the five affected males experienced severe hyperammonemia, with levels for Individual 1 and 2 rising to > 1000 and 1400 μmol/L, respectively. Individual 1's hyperammonemic crisis was precipitated following intense physical activity, which eventually led to their death. Individual 2 presented with a 10‐day progressive history of blurred vision, headaches, loss of coordination, and behavioral changes. He was admitted with encephalopathy and passed away shortly after from a tonsillar herniation. A postmortem diagnosis of OTC deficiency was confirmed by autopsy. Both individuals had been following a self‐imposed vegetarian diet as their only form of management. At least two of the other three affected males have remained asymptomatic.

### 3.2 Functional Impact of the c.‐106C>A Variant

The *Family A Individual* listed in Table 2 is the nephew of the proband, but due to lack of clinical information beyond a liver biopsy, he was excluded from Table 1. The measured OTC activity from his liver biopsy was 8.2 μmol/g liver/min, which was 8.6% of the same day control value (94.9 μmol/g liver/min). The CPS1 activity was 3.3 μmol/g liver/min, with a same day control of 4.6 μmol/g liver/min. The Family B proband's liver OTC activity was 2.2 μmol/g liver/min, which was 3% of the same day control value (73.2 μmol/g liver/min). The CPS1 activity was 5.6 μmol/g liver/min, with a same day control of 3.2 μmol/g liver/min. The Family C Individual, a second cousin of the proband (Individual 2 under Family C in Table 1), had undetectable OTC activity at 0 μmol/g liver/min, indicating a complete loss of function. The same day control was 25–31.7 μmol/g liver/min. CPS1 activity was unavailable; however, arginosuccinate lyase and arginase activity were measured. Arginosuccinate lyase activity was 2.08 μmol/g liver/min, with a normal control value of 3.03 μmol/g liver/min (control range was 2.5–15 μmol/g liver/min). Arginase activity was 48 mmol/h/g liver, with normal controls of 70 and 72 mmol/h/g liver (control range was 10–95 mmol/h/g liver).

**TABLE 2:** Liver OTC enzymatic data in affected males with the c.‐106C>A *OTC* variant from Families A–C.

	OTC activity	
	Measured	Same day control
Family A individual	8.2	94.9 (μmol/g liver/min)
Family B proband	2.2	73.2 (μmol/g liver/min)
Family C individual	0	25–31.7 (μmol/g liver/min)

Abbreviations: g = grams, min = minute, OTC = ornithine transcarbamylase.

## 4 Discussion

In this paper we describe and highlight the physiological consequences of the rare c.‐106C>A variant in the promoter region of *OTC* and its association with late‐onset OTC deficiency. The biochemical and clinical variability was highlighted amongst affected males from three independent pedigrees, with our findings supporting the pathogenicity of the c.‐106C>A variant. Currently, this variant has five entries in the ClinVar database, four classified as “pathogenic” and one classified as “likely pathogenic.” The *OTC* c.‐106C>A variant is not identified on the gnomeAD (accessed July 27, 2025 version 4.1.0).

Three prior studies have examined the c.‐106C>A variant in the context of late‐onset OTC deficiency. Jang et al. sequenced the conserved promoter and enhancer regions of the *OTC* gene from nine unrelated males who were suspected to have OTC deficiency; however, no variants were identified within the coding regions or intron splice sites [10]. Eight of the nine males possessed a sequence variant in the promoter region of the *OTC* gene, while one male possessed a variant located in the upstream enhancer region [10]. The variant presented in our study, c.‐106C>A, was one of five found to be located within a known or predicted transcription factor binding site, and functional testing showed decreased reporter gene expression and decreased binding of hepatocyte nuclear factor 4 (HNF‐4) to the *OTC* promoter [10]. Han et al. further demonstrated the segregation of the c.‐106C>A variant in two independent families. Their findings also confirmed reduced promoter activity via luciferase assays, supporting its pathogenicity under metabolic stress. These findings help explain the markedly reduced OTC enzyme activity presented in our study (Table 2), as the c.‐106C>A variant appears to impair *OTC* expression. The reduced expression levels are likely due to disrupted binding of relevant transcription factors, such as HNF‐4, which is known to activate *OTC* transcription [14].

No residual OTC activity was detected in the Family C Individual (Table 2), suggesting complete loss of enzyme activity can occur with this variant. However, additional cases are needed to confirm this observation. Although sampling error cannot be entirely excluded, normal ASL and arginase activity levels from the same specimen argue against sample degradation or a technical artifact. The variable expressivity of the c.‐106C>A variant is likely influenced by environmental factors. In both families A and B, the probands experienced environmental stressors that contributed to their hyperammonemic episodes. During the second presentation for the family A proband, he had concurrent gastroenteritis that impaired medication adherence and precipitated a hyperammonemia crisis. For the family B proband, valproate use and physical exertion were contributory factors in his initial presentation. In contrast, other members of the families with the variant did not experience similar hyperammonemic crises, which can in part be attributed to more favorable environmental factors that do not necessarily play a role in exacerbating hyperammonemia.

Hertzog et al. identified three individuals with the c.‐106C>A variant, providing further biochemical and clinical data to the limited literature on the c.‐106C>A variant [6]. Similar to the probands from Families A and B in our study, these individuals had no coding or splice‐site variants identified [6]. Only through targeted sequencing of regulatory regions in *OTC* was a variant confirmed, highlighting the importance of evaluating noncoding regions in patients with suspected OTC deficiency when initial attempts at sequencing fail to identify any variants [6]. Patient 1 in Hertzog et al.'s study was asymptomatic but underwent genetic testing due to a half‐sibling being diagnosed with OTC deficiency at age 12 who later died at age 33 from hyperammonic encephalopathy [6]. In contrast, Patient 2 presented at 5 years old after emesis for numerous weeks, and Patient 3 also presented at 5 years old with an 18‐month history of emesis, lethargy, and abnormal behavior [6]. These presentations highlight the clinical variability associated with the c.‐106C>A variant, which is consistent with the observed heterogeneity in the affected males we present.

At the time of his presentation with hyperammonemic encephalopathy, the proband of Family B in our study was receiving valproate [3]. There have been several documented reports of encephalopathy among individuals with OTC deficiency while receiving valproate [3, 15, 16, 17]. Valproate administration has also been shown to trigger acute hyperammonemia in patients with no underlying metabolic issues, and to unmask OTC deficiency in heterozygous females [18, 19, 20]. Although previously attributed to inhibition of carbamoyl phosphate synthetase, recent research suggests that valproate induces hyperammonemia through inhibition of N‐acetylglutamate synthase by valproyl‐CoA, a metabolite of valproate [21, 22]. Given this, patients with OTC deficiency should be monitored closely when prescribed valproate, as it may precipitate or exacerbate hyperammonemic episodes.

This study adds to the limited literature on the c.‐106C>A promoter region variant, strengthening its association with late‐onset OTC deficiency and supporting its pathogenic potential. By integrating biochemical, clinical, and enzymatic activity data with existing studies, we provide further support for how this variant may contribute to disease, although additional functional characterization is needed to further elucidate its pathogenic mechanism.

## Author Contributions

**Samuel Quinn Tholl:** concept and design, analysis and interpretation of data, drafting the article or revising it critically for important intellectual content. **Wendy McCaul:** performing the molecular studies and interpretation of data. **Anthony Rupar:** concept and design, analysis and interpretation of data, drafting the article or revising it critically for important intellectual content. **Melanie P. Napier:** collecting the clinical information and revising the article for important intellectual content. **Natalya Karp:** collecting the clinical information and revising the article for important intellectual content. **Andrea C. Yu:** concept and design, analysis and interpretation of data, drafting the article or revising it critically for important intellectual content. **Suzanne Ratko:** collecting the clinical information and revising the article for important intellectual content. **Aneal Khan:** drafting the article or revising it critically for important intellectual content. **Michael Geraghty:** drafting the article or revising it critically for important intellectual content. **Chitra Prasad:** concept and design, analysis and interpretation of data, drafting the article or revising it critically for important intellectual content.

## Funding

This work was supported by grant #2315 issued by the Children's Hospital Foundation (Children's Hospital, London Health Sciences Center, London, Ontario, Canada). The authors confirm that the funding source has no role in the study design, data collection, analysis, or writing of the manuscript.

## Ethics Statement

Ethics approval was obtained from Western University via Western University Ethics Manager (WREM), with study number 116586. Study approval was also obtained by Lawson Health Research Institute.

## Consent

Consent was deemed not required for this retrospective study by our institutional ethics board. All individuals in the study are referred to as a pseudonym (i.e., Individual 1), and in no way is it possible to identify any individual based on their data contained in the study. Although identifying information was collected, it is minimal. This was deemed acceptable according to our institutional ethics board and was approved with the initial ethics application.

## Conflicts of Interest

Melanie Napier is an employee of and may hold stock in GeneDx. The other authors declare no conflicts of interest.

## Data Availability

The data that support the findings of this study are available from the corresponding author upon reasonable request.

## References

[1] Orphanet , Ornithine Transcarbamylase Deficiency (Orphanet, 2024), https://www.orpha.net/en/disease/detail/664.

[2] U. Lichter‐Konecki , L. Caldovic , H. Morizono , K. Simpson , N. A. Mew , and E. MacLeod , “Ornithine Transcarbamylase Deficiency,” in GeneReviews, ed. M. P. Adam , S. Bick , G. M. Mirzaa , R. A. Pagon , S. E. Wallace , and A. Amemiya (University of Washington, 2013), https://www.ncbi.nlm.nih.gov/books/NBK154378/.

[3] V. Thakur , A. Rupar , D. Ramsay , et al., “Fatal Cerebral Edema From Late‐Onset Ornithine Transcarbamylase Deficiency in a Juvenile Male Patient Receiving Valproic Acid,” Pediatric Critical Care Medicine 7, no. 3 (2006): 273–276. 16575347 10.1097/01.PCC.0000216682.56067.23

[4] E. T. Rush , J. E. Hartmann , J. C. Skrabal , and W. B. Rizzo , “Late‐Onset Ornithine Transcarbamylase Deficiency: An Under Recognized Cause of Metabolic Encephalopathy,” SAGE Open Medical Case Reports 2 (2014): 2050313X14546348. 10.1177/2050313X14546348 PMC4857352 27489649

[5] J. H. Choi , B. H. Lee , J. H. Kim , et al., “Clinical Outcomes and the Mutation Spectrum of the *OTC* Gene in Patients With Ornithine Transcarbamylase Deficiency,” Journal of Human Genetics 60 (2015): 501–507. 25994866 10.1038/jhg.2015.54

[6] A. Hertzog , A. Selvanathan , R. Halligan , et al., “A Serendipitous Journey to a Promoter Variant: The c.‐106C>A Variant and Its Role in Late‐Onset Ornithine Transcarbamylase Deficiency,” JIMD Reports 63 (2022): 271–275. 35822098 10.1002/jmd2.12289 PMC9259394

[7] M. Tuchman , N. Jaleel , H. Morizono , et al., “Mutations and Polymorphisms in the Human Ornithine Transcarbamylse Gene,” Human Mutation 19, no. 93 (2002): 93–107. 11793468 10.1002/humu.10035

[8] S. Yamaguchi , L. L. Brailey , H. Morizono , A. E. Bale , and M. Tuchman , “Mutations and Polymorphisms in the Human Ornithine Transcarbamylase (*OTC*) Gene,” Human Mutation 27, no. 7 (2006): 626–632. 16786505 10.1002/humu.20339

[9] A. Brassier , S. Gobin , J. B. Arnoux , et al., “Long‐Term Outcomes in Ornithine Transcarbamylase Deficiency: A Series of 90 Patients,” Orphanet Journal of Rare Diseases 10 (2015): 58. 25958381 10.1186/s13023-015-0266-1 PMC4443534

[10] Y. J. Jang , A. L. LaBella , T. P. Feeney , et al., “Disease‐Causing Mutations in the Promoter and Enhancer of the Ornithine Transcarbamylase Gene,” Human Mutation 39, no. 4 (2018): 527–536. 29282796 10.1002/humu.23394 PMC7388160

[11] C. L. Pridmore , J. T. R. Clark , and S. Blaser , “Ornitine Transcarbamylase Deficiency in Females: An Often‐Overlooked Cause of Treatable Encephalopathy,” Journal of Child Neurology 10, no. 5 (1995): 369–374. 7499756 10.1177/088307389501000506

[12] K. Sen , R. Izem , Y. Long , et al., “Are Asymptomatic Carriers of OTC Deficiency Always Asymptomatic? A Multicentric Retrospective Study of Risk Using the UCDC Longitudinal Study Database,” Molecular Genetics & Genomic Medicine 12, no. 5 (2024): e2443. 38634223 10.1002/mgg3.2443 PMC11024633

[13] S. T. Han , K. J. Anderson , H. T. Bjornsson , et al., “A Promoter Variant in the *OTC* Gene Associated With Late and Variable Age of Onset Hyperammonemia,” JIMD Reports 63, no. 1 (2022): 80–89. 35605046 10.1002/jimd.12524 PMC10323875

[14] A. Kimura , A. Nishiyori , T. Murakami , et al., “Chicken Ovalbumin Upstream Promoter‐Transcription Factor (COUP‐TF) Represses Transcription From the Promoter of the Gene for Ornithine Transcarbamylase in a Manner Antagonistic to Hepatocyte Nuclear Factor‐4 (HNF‐4),” Journal of Biological Chemistry 268, no. 15 (1993): 11125–11133. 8496174

[15] S. Mehta , S. Tayabali , and R. Lachmann , “Valproate‐Induced Hyperammonemia – Uncovering an Underlying Inherited Metabolic Disorder: A Case Report,” Journal of Medical Case Reports 12 (2018): 134. 29769109 10.1186/s13256-018-1666-3 PMC5956736

[16] M. Oechsner , C. Steen , H. J. Stürenburg , and A. Kohlschutter , “Hyperammonaemic Encephalopathy After Initiation of Valproate Therapy in Unrecognized Ornithine Transcarbamylase Deficiency,” Journal of Neurology, Neurosurgery & Psychiatry 64 (1998): 680–682. 9598692 10.1136/jnnp.64.5.680 PMC2170080

[17] D. Kazmierski , N. Sharma , K. O'Leary , and P. Ochieng , “Valproate‐Induced Fatal Acute Hyperammonaemia‐Related Encephalopathy in Late‐Onset Ornithine Transcarbamylase Deficiency,” BMJ 14, no. 5 (2021): e241429. 10.1136/bcr-2020-241429 PMC8154925 34035022

[18] B. E. Gidal , C. M. Inglese , J. F. Meyer , M. E. Pitterle , J. Antonopolous , and R. S. Rust , “Diet‐ and Valproate‐Induced Transient Hyperammonemia: Effect of L‐Carnitine,” Pediatric Neurology 16 (1997): 301–305. 9258962 10.1016/s0887-8994(97)00026-x

[19] Y. Sattar , B. Merotto , A. Dedousis , M. Aadil , and A. Zil‐E‐Ali , “Valproic Acid‐Induced Hyperammonemia With Encephalopathy (VIHE): A Case Report,” Journal of Medical Research and Innovation 2, no. 1 (2018): e000108.

[20] D. Honeycutt , K. Callahan , L. Rutledge , and B. Evans , “Heterozygote Ornithine Transcarbamylase Deficiency Presenting as Symptomatic Hyperammonemia During Initiation of Valproate Therapy,” Neurology 42 (1992): 666–668. 1549234 10.1212/wnl.42.3.666

[21] J. Duarte , S. Macias , F. Coria , E. Fernandez , and L. E. Clavería , “Valproate ‐ Induced Coma: Case Report and Literature Review,” Annals of Pharmacotherapy 27 (1993): 582–583. 8347908 10.1177/106002809302700510

[22] C. C. P. Aires , A. Cruchtten , L. IJkst , et al., “New Insights on the Mechanisms of Valproate‐Induced Hyperammonemia: Inhibition of Hepatic N‐Acetylglutamate Synthase Activity by Valproyl‐CoA,” Journal of Hepatology 55, no. 2 (2010): 426–434. 21147182 10.1016/j.jhep.2010.11.031

